# Colorectal cancer stem cell-derived exosomal long intergenic noncoding RNA 01315 (LINC01315) promotes proliferation, migration, and stemness of colorectal cancer cells

**DOI:** 10.1080/21655979.2022.2065800

**Published:** 2022-04-26

**Authors:** Youran Li, Minna Wu, Shanshan Xu, Hua Huang, Lei Yan, Yunfei Gu

**Affiliations:** a Department of Colorectal Surgery, Jiangsu Province Hospital of Chinese Medicine, Affiliated Hospital of Nanjing University of Chinese Medicine, Nanjing, Jiangsu, China; b Department of Anorectal, Changshu Hospital Affiliated to Nanjing University of Chinese Medicine, Changshu, Suzhou, Jiangsu, China

**Keywords:** Colorectal cancer, stem-like cell, exosomal, LINC01315, stemness

## Abstract

The effect of long intergenic noncoding RNA 01315 (LINC01315) on colorectal cancer has widely been proved. Nevertheless, how LINC01315 functions in the stemness of colorectal cancer and whether LINC01315 exists in colorectal cancer stem-like cell-derived exosomes remain dim, which are thus investigated in this research. CD133^+^/CD44^+^ colorectal cancer stem cells were sorted and verified through flow cytometry. Exosomes derived from CD133^+^/CD44^+^ colorectal cancer stem cells were collected. The viability, proliferation, stemness and migration of CD133^+^/CD44^+^, CD133^−^/CD44^−^, and colorectal cancer cells after transfection or the co-culture with exosomes were detected by MTT, colony formation, spheroid, and wound healing assays, respectively. Expressions of LINC01315, BCL-2, Bax, cleaved caspase-3, MMP-9, E-cadherin, and vimentin in cells or exosomes were analyzed using western blot or qRT-PCR. Genes interacted with LINC01315 in colorectal cancer were predicted by bioinformatics analysis. The results showed that LINC01315 was high-expressed in CD133^+^/CD44^+^ colorectal cancer stem cells and exosomes. Compared with colorectal cancer cells, the viability, proliferation, stemness, and migration of CD133^+^/CD44^+^ cancer cells were stronger, while these of CD133^−^/CD44^−^ cancer cells were weaker. Besides, LINC01315 silencing decreased the viability, proliferation, stemness, and migration of CD133^+^/CD44^+^ cancer cells, while sh-LINC01315 inhibited the promotive effects of CD133^+^/CD44^+^ cancer cell-derived exosomes on the viability, proliferation, stemness, and migration of colorectal cancer cells. LINC01315 was also found to be correlated with DPEP1, KRT23, ASCL2, AXIN2, and DUSP4 in colorectal cancer. In conclusion, colorectal cancer stem cell-derived exosomal LINC01315 promotes the proliferation, migration, and stemness of colorectal cancer cells.

## GRAPHICAL ABSTRACT

**Figure:**
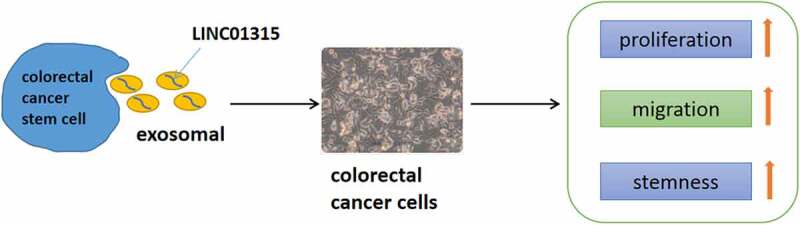

## Highlights

- LINC01315 is highly expressed in CD133+/CD44+ colorectal cancer cells
- LINC01315 silencing inhibits the stemness of CD133+/CD44+ colorectal cancer cells
- LINC01315 is highly expressed in CD133+/CD44+ colorectal cancer cell-derived exosomes
- Colorectal cancer stem cell-derived exosomal LINC01315 promotes proliferation, migration, and stemness of colorectal cancer cells

## Introduction

Colorectal cancer is one of the most common malignant tumors and leading causes of cancer-related deaths in the world. According to the statistics from 2020 global cancer survey, colorectal cancer ranks third in terms of incidence, and second in the aspect of mortality [1]. Currently, surgical resection, chemotherapy, and radiotherapy are the main treatment options of colorectal cancer, among which patients with early cancer are given priority to surgical resection, while those with advanced colorectal cancer commonly receive radiotherapy and chemotherapy [2–4]. However, the drug resistance of cancer cells after radiotherapy and chemotherapy as well as the recurrence and metastasis of cancer after surgery still seriously affect the prognosis of patients. Therefore, it is of great importance to seek highly specific molecular targets for the treatment of colorectal cancer.

LncRNA is a set of transcripts longer than 200 nucleotides, which cannot encode proteins, but can regulate gene expression at the epigenetic, transcriptional, and post-transcriptional levels [5]. Besides, lncRNA is closely associated with the development of assorted cancers including colorectal cancer [6,7]. LINC01315 is a newly recognized lncRNA and its expression has been discovered to be elevated in cancers such as thyroid cancer, oral squamous cell carcinoma, and breast cancer [8–10]. Furthermore, LINC01315 is proven to be highly expressed in colorectal cancer and LINC01315 silencing suppresses the aggressive phenotypes of colorectal cancer cells [11]. Nevertheless, the effect of LINC01315 on colorectal cancer and its detailed mechanism require to be further elucidated.

The occurrence and maintenance of cancers largely depend on a small fraction of cancer cells, namely, called cancer stem cells, as they have unlimited self-renewal capacity and the potential to induce tumorigenesis and drug resistance in chemotherapy [12,13]. Application of conventional anti-cancer drugs has a short-term effect on the proliferation of tumor cells, but this treatment cannot eradicate the highly cancerous tumor stem cells, making it difficult to kill tumor cells with standard therapeutics and leading to the recurrence of cancer [14]. Besides, the stemness of cancer cells has been demonstrated to be regulated by lncRNAs. For example, lncRNA HAND2-AS1 enhances the self-renewal ability of liver cancer stem cells [15], and lncRNA HOTTIP modulates the properties of pancreatic cancer stem cells by regulating HOXA9 level [16]. Therefore, we wonder whether the effects of LINC01315 on colorectal cancer cells are associated with colorectal cancer stem cells. RNA molecules, including mRNA, miRNA, circRNA, as well as lncRNA, are highly enriched in exosomes, which are tiny vesicles (30–150 nm) secreted by most cell types [17]. Moreover, carcinoma-associated fibroblasts-derived exosomal lncRNA H19 has been found to promote the stemness and chemoresistance of colorectal cancer cells. Hence, we strongly want to know whether colorectal cancer stem cell-derived exosomes can secrete LINC01315 to promote the development of colorectal cancer.

Therefore, in this research, we sorted colorectal cancer stem cells and collected the exosomes derived from cancer stem cells, aiming to fathom out the role of LINC01315 in colorectal cancer stem cells, and the effects of colorectal cancer stem cell-derived exosomal LINC01315 on the biological function of colorectal cancer.

## Material and methods

### Cell culture

Human colorectal cancer cell lines SW480 (CL-0223) and HCT116 (CL-0096) were purchased from Procell (Wuhan, China). SW480 cells were grown in Leibovitz’s L-15 medium (PM151010, Procell) containing 10% fetal bovine serum (FBS; 164210–500, Procell) and 1% penicillin-streptomycin solution (P/S; PB180120, Procell), and HCT116 cells were cultured in McCoy’s 5A medium (PM150710, Procell) supplemented with 10% FBS and 1% P/S. All cells were incubated under the Heracell 240i CO_2_ Incubator (Thermo Scientific, Waltham, Massachusetts, USA).

### Flow cytometry

Flow cytometry was used to sort out CD133^+^/CD44^+^ SW480 and CD133^+^/CD44^+^ HCT116 cells as previously reported [14]. In brief, SW480 and HCT116 cells were collected and incubated with phycoerythrin (PE)-conjugated CD133 antibody (ab253262, Abcam, Cambridge, UK) and fluorescein isothiocyanate (FITC)-conjugated CD44 antibody (ab30405, Abcam). Finally, the CD133^+^/CD44^+^ SW480 cells, CD133^+^/CD44^+^ HCT116 cells, CD133^−^/CD44^−^ SW480 cells and CD133^−^/CD44^−^ HCT116 cells were sorted under the help of Attune NxT flow cytometer (Thermo Scientific).

### Transfection

Short-hairpin RNA (shRNA) against LINC01315 (sh-LINC01315#1, sh-LINC01315#2) and negative control for shRNA (shNC) were synthesized by RIBOBIO (Guangzhou, China). In a nutshell, CD133^+^/CD44^+^ SW480 and CD133^+^/CD44^+^ HCT116 cells were separately seeded in a 6-well plate. After the cell confluence reached about 70%, sh-LINC01315#1, sh-LINC01315#2 and shNC were transfected into the cells through the Lipo6000 transfection reagent (C0526, Beyotime, Shanghai, China). Forty-eight hours (h) after transfection, the cells were collected for later use.

### Isolation, observation and co-culture of exosomes

Exosomes derived from transfected or untransfected SW480 and HCT116 cells were isolated in the light of the previous literatures [18,19]. Briefly, after cells reached about 85% confluence, the original culture medium was removed and refreshed with the FBS-free medium. After the cells were further cultured for 48 h, the cell suspensions were harvested and then centrifuged at 300 × *g* for 10 minutes (min) to remove the cell debris. Next, the supernatant was collected and centrifuged at 2000 × *g* for 10 min to remove dead cells, followed by being successively centrifuged at 10,000 × *g* for 30 min and at 100,000 × *g* for 70 min. Then, the precipitate was gathered, re-suspended with 200 μl phosphate buffer solution (PBS; PB180327, Procell), and centrifuged at 100,000 × *g* for 70 min. Later, the exosome pellets were re-suspended with 200 μl PBS, subsequent to which their concentrations were quantified with a BCA protein quantification kit (MA0082, Meilunbio, Dalian, China). A transmission electron microscope (HT7700, Beijing SJC Science and Trade Co., Ltd., Beijing, China) was used to observe whether the exosomes isolated from SW480 and HCT116 cells were transfected or untransfected. For exosomal co-culture [20], 10 µg exosomes were added into the cultured medium of SW480 and HCT116 cells to incubate the cells for 24 h. The cells after incubation were harvested for later use.

### Quantitative real-time PCR (qRT-PCR)

The expression of LINC01315 in SW480 cells and HCT116 cells, CD133^+^/CD44^+^ SW480 cells, and CD133^+^/CD44^+^ HCT116 cells, and exosomes isolated from SW480 and HCT116 cells was quantified through qRT-PCR. In brief, the total RNA in the above-mentioned samples was isolated using a total RNA extraction kit (19201ES60, Yeasen, Shanghai, China). After the concentration of isolated RNA was detected using a UV spectrophotometer (NanoDrop, Thermo Scientific), the cDNA was synthesized using the RNA with the help of a cDNA synthesis kit (11119ES60, Yeasen). The cDNA was subsequently mixed with SYBR Green Master Mix (11203ES03, Yeasen), and amplified using QuantStudio 7 System (Applied Biosystems, Waltham, Massachusetts, USA) under the following condition: pre-denaturation at 95°C for 5 min; denaturation at 95°C for 10 s (s); annealing at 56°C for 20 s; elongation at 72°C for 20 s. The process from denaturation to elongation was repeated for 40 cycles. The sequences of forward (F) and reverse (R) primers were listed below: LINC01315 (F: 5'-ACATTGAGCCTGCTCCTGTC-3', R: 5'-TTTCCGGTAGGGGGAAAACG-3'); glyceraldehyde-3-phosphate dehydrogenase (GAPDH; F: 5'-GGGCTGCTTTTAACTCTGGT-3', R: 5'-GCAGGTTTTTCTAGACGG-3').

### Fluorescent labeling and transfer validation of exosomes

Fluorescent labeling and transfer validation of exosomes were performed according to the existing research [19]. At first, the exosomes were labeled with PKH67 Green Fluorescent Cell Linker Kit (PKH67GL, Merck, St. Louis, Missouri, USA) in light of the manufacturer’s protocols. Then, the labeled exosomes were incubated with SW480 cells for 24 h, and the cells were also stained with 4’,6-diamidino-2-phenylindole (DAPI; P36931, Invitrogen, Waltham, Massachusetts, USA) for 30 min. Finally, the cells and exosomes were observed by a fluorescence microscope (DM2500; Leica, Wetzlar, Germany) and photographed at a magnification of × 400.

### MTT assay

The viability of cells after being cultured for 24 h, 48 h, and 72 h was determined by MTT assay. Concretely, 6 × 10^3^ cells were seeded into each well of the 96-well plates and cultured for 24 h, 48 h, and 72 h. Then, 50 μl 1× MTT buffer (KGA311, KeyGEN BioTECH, Nanjing, China) was added to each well to further incubate the cells for 4 h. After the medium in each well was removed, 150 μl of dimethyl sulfoxide (DMSO; KGT5131, KeyGEN BioTECH) was put into each well, subsequent to which the optical density (OD) value at a wavelength of 490 nm was detected by a Varioskan LUX Microplate reader (Thermo Scientific).

### Colony formation assay

The proliferative ability of cells was evaluated using colony formation assay. Briefly, 1 × 10^3^ cells were collected and added into each well of 6-well plates. Then, the cells were cultured for 14 days, and the medium was refreshed every 2 days. Next, the culture medium was removed and the cells were fixed by 4% paraformaldehyde (P0099, Beyotime, Shanghai, China)for 10 min. After being washed twice with PBS, the cells were further stained by 1× crystal violet staining solution (KGA229, KeyGEN BioTECH) for 15 min. Finally, the stained cells were rinsed three times by PBS and further photographed by a C-LUX camera (Leica).

### Spheroid assay

Sphere formation ability of cells was assessed using spheroid assays according to the literature of Asadzadeh et al. [21]. Firstly, cell culture medium containing 10% Matrigel (354,234, Corning, Corning, New York, USA) was added into each well of the 96-well plates. Then, 2.5 × 10^3^ cells were further added into each well and incubated for 10 days. Finally, the image of spheres in each well was observed under DMi8 S optical microscope (Leica) and captured at a magnification of × 200.

### Wound healing assay

The migratory ability of cells was analyzed using wound healing assay. In a nutshell, the cell culture insert (81,176, Ibidi, Martin Reid, Germany) was first placed into the 6-well plates. Next, cells were added into the 6-well plates. After cell confluence reached about 100%, the insert of the cell culture was removed and the culture medium was refreshed with an FBS-free medium. Then, the cells were further cultivated for 48 h, followed by the removal of cell culture insert from each well. Ultimately, the cell images at 0 h and 48 h were observed by an optical microscope and captured at a magnification of × 100.

### Western blot assay

The related protein expression levels in SW480 cells and HCT116 cells with or without transfection, CD133^+^/CD44^+^ SW480 cells, CD133^+^/CD44^+^ HCT116 cells, CD133^−^/CD44^−^ SW480 cells, CD133^−^/CD44^−^ HCT116 cells, and exosomes isolated from SW480 and HCT116 cells were quantified through Western blot assay. In brief, total protein in the above samples was firstly isolated using Total Protein Extraction Cell Lysis Buffer (V900854, Merck). After the concentration of protein was determined using a QuantiPro BCA Detection Kit (QPBCA, Merck), the protein was denatured with protein loading buffer (MA0003-D, Meilunbio) at 100°C for 5 min. Subsequently, the protein samples were separated by sodium dodecyl sulfate-polyacrylamide gel electrophoresis (SDS-PAGE; MA0159, Meilunbio). Afterward, the separated protein was transferred onto polyvinylidene fluoride (PVDF) membranes (KGP114-1, KenGEN BioTECH), followed by a further incubation with Western blocking buffer (MA0097, Meilunbio) for 1 h. Later, the membranes were incubated with primary antibodies at 4°C for 16 h and then cultivated with corresponding secondary antibodies at room temperature for 1 h. At last, the protein band was visualized under Image Lab 3.0 Imager (Bio-Rad, Hercules, California, USA) using the Immobilon ECL Ultra Western HRP Substrate (WBULS0100, Merck). The primary antibodies adopted in this research mainly included those against Oct-4 (1:5000, ab200834, Abcam), Prominin (1:1000, #86781, Cell Signaling Technology, Boston, Massachusetts, USA), SOX2 (1:2000, ab92494, Abcam), CD9 (1:1000, ab236630, Abcam), CD63 (1:4000, ab134045, Abcam), HSP70 (1:1000, ab2787, Abcam), BCL-2 (1:1000, ab32124, Abcam), Bax (1:3000, ab32503, Abcam), cleaved caspase-3 (1:500, ab32042, Abcam), E-cadherin (1:500, ab40772, Abcam), MMP-9 (1:6000, ab76003, Abcam), Vimentin (1:1000, ab20346, Abcam) and GAPDH (1:10000, ab8245, Abcam), and the secondary antibodies applied were goat-anti rabbit IgG (1:20000, ab6721, Abcam) and goat-anti mouse IgG (1:10000, ab6789, Abcam).

### Bioinformatics analysis

Website of ciBioportal (http://www.cbioportal.org/) was used to analyze genes co-expressed with LINC01315. GEO analysis (GSE23878 data set) was employed to analyze the differentially expressed genes in colorectal cancer. Website of jvenn (http://jvenn.toulouse.inra.fr/app/example.html) was utilized to intersect the common genes in the two data above.

### Statistical analysis

GraphPad prism 8.0 was applied to analyze the data in the current research. An independent sample *t* test was conducted to compare the data from two groups. Two-way analysis of variance (ANOVA) was employed to analyze the data in MTT assays. One-way ANOVA was utilized to contrast the data from more than two groups. Final statistical data were shown in the form of mean ± standard deviation (SD). *P* < 0.05, *P* < 0.01, and *P* < 0.001 were perceived as the statistical difference.

## Results

In this study, we explored the role of LINC01315 in colorectal cancer stem cells, and the effects of colorectal cancer stem cell-derived exosomal LINC01315 on the biological function of colorectal cancer. To be specific, colorectal cancer stem cells were sorted and the exosomes derived from cancer stem cells were collected. LINC01315 was silenced or overexpressed in CD133+ CD44+ colorectal cancer stem cells, and exosomes were extracted to detect the effects of exosomes on SW480 and HCT116 cells. As a result, CD133+/CD44+ colorectal cancer stem cell-derived exosomal LINC01315 was found to promote the proliferation, migration, and stemness of colorectal cancer cells.

## LINC01315 was highly expressed in CD133^+^/CD44^+^ colorectal cancer cells

Considering that CD133 and CD44 are the main markers of tumor-initiating cell subpopulation from SW480 and HCT116 cells [14], we sorted CD133^+^/CD44^+^ SW480 and CD133^+^/CD44^+^ HCT116 cells using flow cytometry (Supplementary Figure S1(a)). Subsequently, other markers, including Oct-4, Prominin, and SOX2 in the CD133^+^/CD44^+^ SW480 and CD133^+^/CD44^+^ HCT116 cells, were also determined by western blot. As depicted in Supplementary Figure S1(b–d), the levels of Oct-4, Prominin, and SOX2 were all up-regulated in CD133^+^/CD44^+^ colorectal cancer cells when compared with those in the CD133^−^/CD44^−^ colorectal cancer cells (*P* < 0.001).

## CD133^+^/CD44^+^ colorectal cancer cells had higher viability and stronger abilities of proliferation, sphere formation, and migration, while CD133^−^/CD44^−^ colorectal cancer cells exerted the opposite effects

As shown in Supplementary Figure S1(e–f), the viabilities of CD133^−^/CD44^−^ SW480 cells and CD133^−^/CD44^−^ HCT116 cells at 48 h and 72 h were lower (*P* < 0.05), while the viabilities of CD133^+^/CD44^+^ SW480 cells and CD133^+^/CD44^+^ HCT116 cells at 48 h and 72 h were higher than those of corresponding SW480 and HCT116 cells (*P* < 0.001). Meanwhile, compared with SW480 and HCT116 cells, the proliferation rate (Supplementary Figure S1(g–j)) and migration rate (Supplementary Figure S1(m–p)) were lower in CD133^−^/CD44^−^ SW480 cells and CD133^−^/CD44^−^ HCT116 cells (*P* < 0.05) but were higher in CD133^+^/CD44^+^ SW480 cells and CD133^+^/CD44^+^ HCT116 cells (*P* < 0.001). In addition, as depicted in Supplementary Figure S1(k–l), there were almost no sphere formation abilities of CD133^−^/CD44^−^ SW480 cells and CD133^−^/CD44^−^ HCT116 cells, while the sphere formation abilities of CD133^+^/CD44^+^ SW480 cells and CD133^+^/CD44^+^HCT116 cells were stronger than those of SW480 and HCT116 cells.

## LINC01315 silencing inhibited the stemness of CD133^+^/CD44^+^ colorectal cancer cells

**Figure 1.**
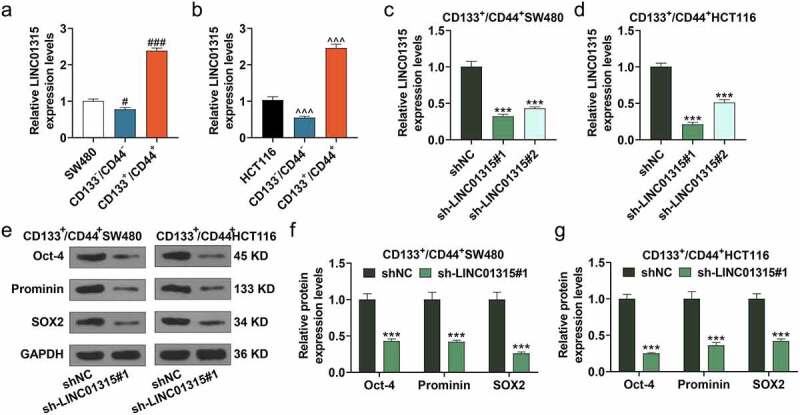
LINC01315 was highly expressed in CD133^+^/CD44^+^ colorectal cancer cells, LINC01315 silencing inhibited the stemness of CD133^+^/CD44^+^ colorectal cancer cells.

**Figure 2.**
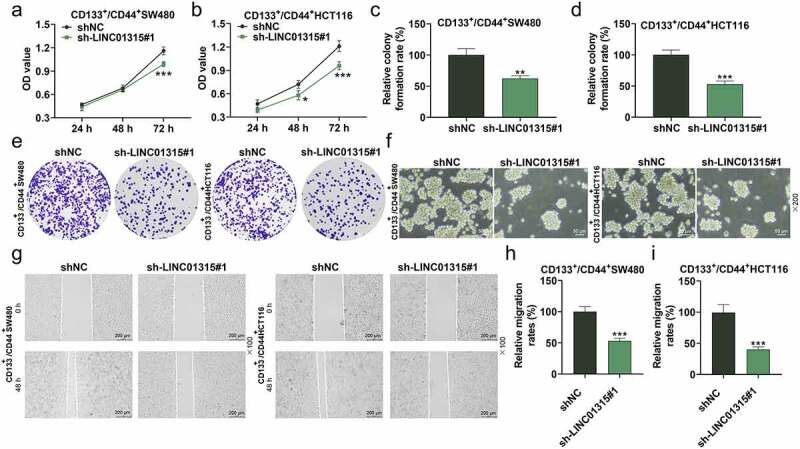
LINC01315 silencing inhibited the stemness of CD133^+^/CD44^+^ colorectal cancer cells.

The expression of LINC01315 was detected in SW480 and HCT116 cells, CD133^−^/CD44^−^ SW480 cells, CD133^−^/CD44^−^ HCT116 cells, CD133^+^/CD44^+^ SW480 cells and CD133^+^/CD44^+^ HCT116 cells. As compared with that in SW480 and HCT116 cells, the expression of LINC01315 was lower in CD133^−^/CD44^−^ colorectal cancer cells (*P* < 0.05) but was higher in CD133^+^/CD44^+^ colorectal cancer cells (*P* < 0.001) (Figure 1(a,b)). To determine the effect of LINC01315 on stemness of colorectal cancer cells, LINC01315 was silenced using shRNA. As exhibited in Figure 1(c,d), sh-LINC01315 down-regulated LINC01315 expression both in CD133^+^/CD44^+^ SW480 cells and CD133^+^/CD44^+^ HCT116 cells (*P* < 0.001), and the silencing efficiency of sh-LINC01315#1 was relatively better than sh-LINC01315#2. Therefore, sh-LINC01315#1 was used in later experiments. Then, we discovered that the expressions of tumor-initiating cell markers encompassing Oct-4, Prominin and SOX2 were all reduced by sh-LINC01315#1 in CD133^+^/CD44^+^ SW480 cells and CD133^+^/CD44^+^ HCT116 cells (*P* < 0.001, Figure 1(e–g)). Furthermore, LINC01315 silencing inhibited the viability (Figure 2(a,b), p < 0.05), proliferation rate (Figure 2(c–e), p < 0.01), sphere formation ability (Figure 2(f)), and migration rate (Figure 2(g–i), *P* < 0.001) of both CD133^+^/CD44^+^ SW480 cells and CD133^+^/CD44^+^ HCT116 cells. All these phenomena verified that LINC01315 silencing inhibited the stemness of CD133^+^/CD44^+^ colorectal cancer cells.

(a-b) The expression of LINC01315 in CD133^+^/CD44^+^ SW480 and CD133^+^/CD44^+^ HCT116 cells was quantified by qRT-PCR. (c-d) After CD133^+^/CD44^+^ SW480 and CD133^+^/CD44^+^ HCT116 cells were transfected with sh-LINC01315#1 and sh-LINC01315#2 for 48 h, the expression of LINC01315 in cells was quantified by qRT-PCR. (e-g) After CD133^+^/CD44^+^ SW480 and CD133^+^/CD44^+^ HCT116 cells were transfected with sh-LINC01315#1 for 48 h, the expressions of Oct-4, Prominin, and SOX2 in cells were measured by western blot. ***P < 0.001, vs. shNC; ^#^*P* < 0.05, ^###^*P* < 0.001, vs. SW480; ^^^^^*P* < 0.001, vs. HCT116 (qRT-PCR: quantitative real-time PCR, shNC: negative control for short-hairpin RNA).

(a-b) The viabilities of CD133^+^/CD44^+^ SW480 and CD133^+^/CD44^+^ HCT116 cells after transfection with sh-LINC01315#1 were detected by MTT assay. (c-e) The proliferative abilities of CD133^+^/CD44^+^ SW480 and CD133^+^/CD44^+^ HCT116 cells following transfection with sh-LINC01315#1 were analyzed by colony formation assay. (F) The sphere formation abilities of CD133^+^/CD44^+^ SW480 and CD133^+^/CD44^+^ HCT116 cells after transfection with sh-LINC01315#1 were evaluated by spheroid assay. (g-i) The migratory abilities of CD133^+^/CD44^+^ SW480 and CD133^+^/CD44^+^ HCT116 cells after transfection with sh-LINC01315#1 were determined by wound healing assay. *P < 0.05, **P < 0.01, ***P < 0.001, vs. shNC (shNC: negative control for short-hairpin RNA, MTT: methyl thiazolyl tetrazolium).

## LINC01315 was highly expressed in exosomes derived from CD133^+^/CD44^+^ colorectal cancer cells

**Figure 3.**
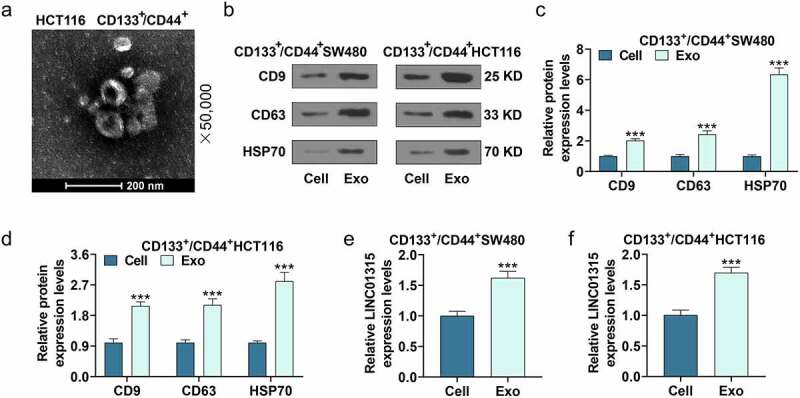
LINC01315 was highly expressed in exosomes derived from CD133^+^/CD44^+^ colorectal cancer cells.

The exosomes derived from CD133^+^/CD44^+^ SW480 cells and CD133^+^/CD44^+^ HCT116 cells were isolated, and the image of CD133^+^/CD44^+^ HCT116 cell-derived exosomes is exhibited in Figure 3(a). Besides, the expressions of exosomal markers including CD9, CD63, and HSP70 were elevated in exosomes as compared with those in the CD133^+^/CD44^+^ SW480 cells and CD133^+^/CD44^+^HCT116 cells (Figure 3(b–d), *P* < 0.001). What is more, LINC01315 expression was discovered to be increased in exosomes as compared with that in the CD133^+^/CD44^+^ SW480 cells and CD133^+^/CD44^+^HCT116 cells (Figure 3(e–f), *P* < 0.001), signifying that the effect of LINC01315 in colorectal cancer cells might be realized through being transferred by exosomes.

(a) The image of CD133^+^/CD44^+^HCT116 cell-derived exosomes was observed by a transmission electron microscope. (b-d) Expressions of CD9, CD63, and HSP70 in CD133^+^/CD44^+^ SW480 cells, CD133^+^/CD44^+^ HCT116 cells, and exosomes derived from the above two cells were quantified using western blot. (e-f) The expression of LINC01315 in CD133^+^/CD44^+^ SW480 cells, CD133^+^/CD44^+^HCT116 cells, and exosomes derived from the above two cells was determined by western blot. ***P < 0.001, vs. Cell. (Exo: exosomes).

## Exosomes derived from CD133^+^/CD44^+^ colorectal cancer cells were absorbed by colorectal cancer cells and they regulated the expression of LINC01315

**Figure 4.**
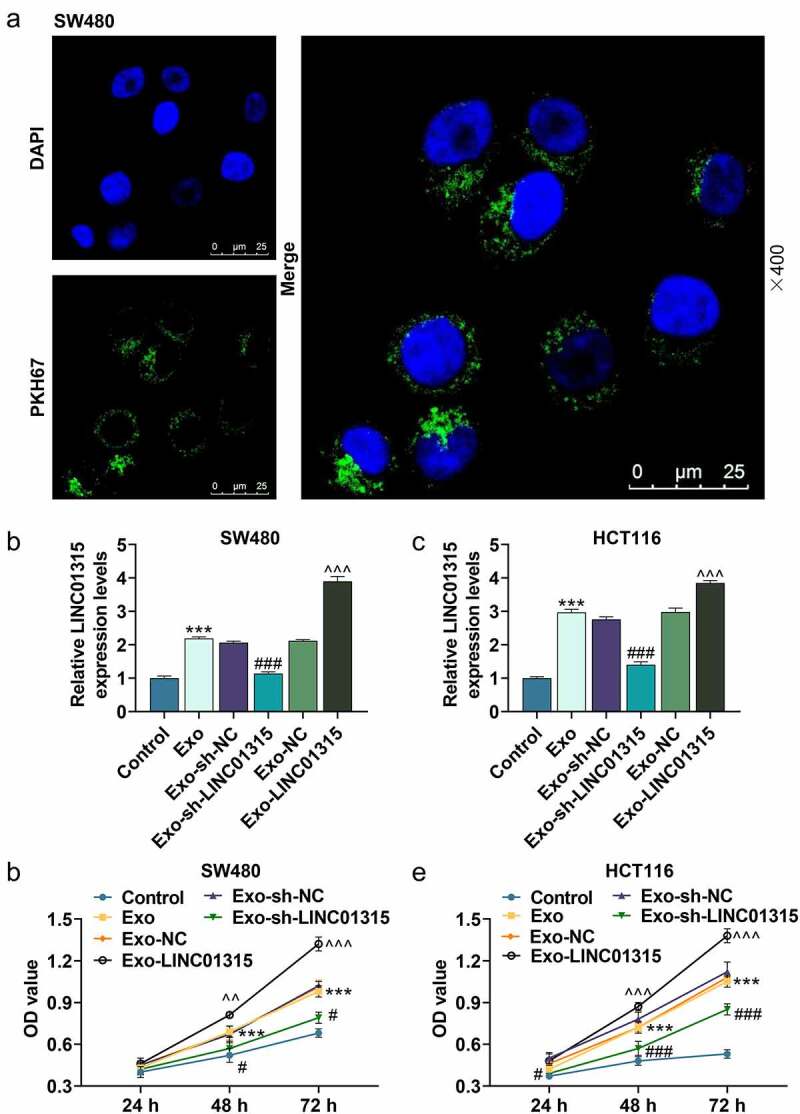
Exosomes derived from CD133^+^/CD44^+^ colorectal cancer cells were absorbed by colorectal cancer cells and they regulated the expression of LINC01315 and viability of colorectal cancer cells.

Therefore, we then verified whether the exosomes could be absorbed by colorectal cancer cells. Initially, SW480 cells were cultured with exosomes derived from CD133^+^/CD44^+^ SW480 cells. As displayed in Figure 4(a), the exosomes were absorbed by SW480 cells. Subsequently, we further collected exosomes derived from CD133^+^/CD44^+^ cells that were transfected with sh-LINC01315 and LINC01315, and then used the exosomes to treat SW480 and HCT116 cells. As illustrated in Figure 4(b,c), the exosomes derived from CD133^+^/CD44^+^ SW480 and CD133^+^/CD44^+^ HCT116 cells boosted LINC01315 expression (*P* < 0.001), and exosomes transfected with LINC01315 promoted LINC01315 expression (*P* < 0.001), while exosomes transfected with sh-LINC01315 inhibited LINC01315 expression (*P* < 0.001).

(a) After SW480 cells were cultured with exosomes derived from CD133^+^/CD44^+^ SW480 cells, the exosomes were absorbed by SW480 cells. (b-c) The expression of LINC01315 in colorectal cancer cells after the co-culture with exosomes derived from corresponding CD133^+^/CD44^+^ colorectal cancer was evaluated by qRT-PCR. (d-e) The viabilities of colorectal cancer cells after the co-culture with exosomes derived from CD133^+^/CD44^+^ colorectal cancer cells were assessed by MTT assay. Control group: SW480 and HCT116 cells were normally cultured for 24 h. Exo group: SW480 and HCT116 cells were cultivated with exosomes derived from corresponding CD133^+^/CD44^+^ SW480 and CD133^+^/CD44^+^ HCT116 cells for 24 h, respectively. Exo-sh-NC group: SW480 and HCT116 cells were incubated with exosomes derived from corresponding CD133^+^/CD44^+^ SW480 and CD133^+^/CD44^+^ HCT116 cells which were transfected with sh-NC for 24 h, respectively. Exo-sh-LINC01315 group: SW480 and HCT116 cells were cultured with exosomes derived from corresponding CD133^+^/CD44^+^ SW480 and CD133^+^/CD44^+^ HCT116 cells which were transfected with sh-LINC01315 for 24 h, respectively. Exo-NC group: SW480 and HCT116 cells were incubated with exosomes derived from corresponding CD133^+^/CD44^+^ SW480 and CD133^+^/CD44^+^ HCT116 cells which were transfected with NC for 24 h, respectively. Exo-LINC01315 group: SW480 and HCT116 cells were cultured with exosomes derived from corresponding CD133^+^/CD44^+^ SW480 and CD133^+^/CD44^+^ HCT116 cells which were transfected with LINC01315 for 24 h, respectively. ***P < 0.001, vs. Control; ^#^*P* < 0.05, ^###^*P* < 0.001, vs. Exo-sh-NC;^^^^*P* < 0.01, ^^^^^*P* < 0.001, vs. Exo-NC (qRT-PCR: quantitative real-time PCR, MTT: methyl thiazolyl tetrazolium, shNC: negative control for short-hairpin RNA, Exo: exosomes).

## Exosomes derived from CD133^+^/CD44^+^ colorectal cancer cells promoted stemness of colorectal cancer cells, while exosomes with down-regulation of LINC01315 reversed this effect

**Figure 5.**
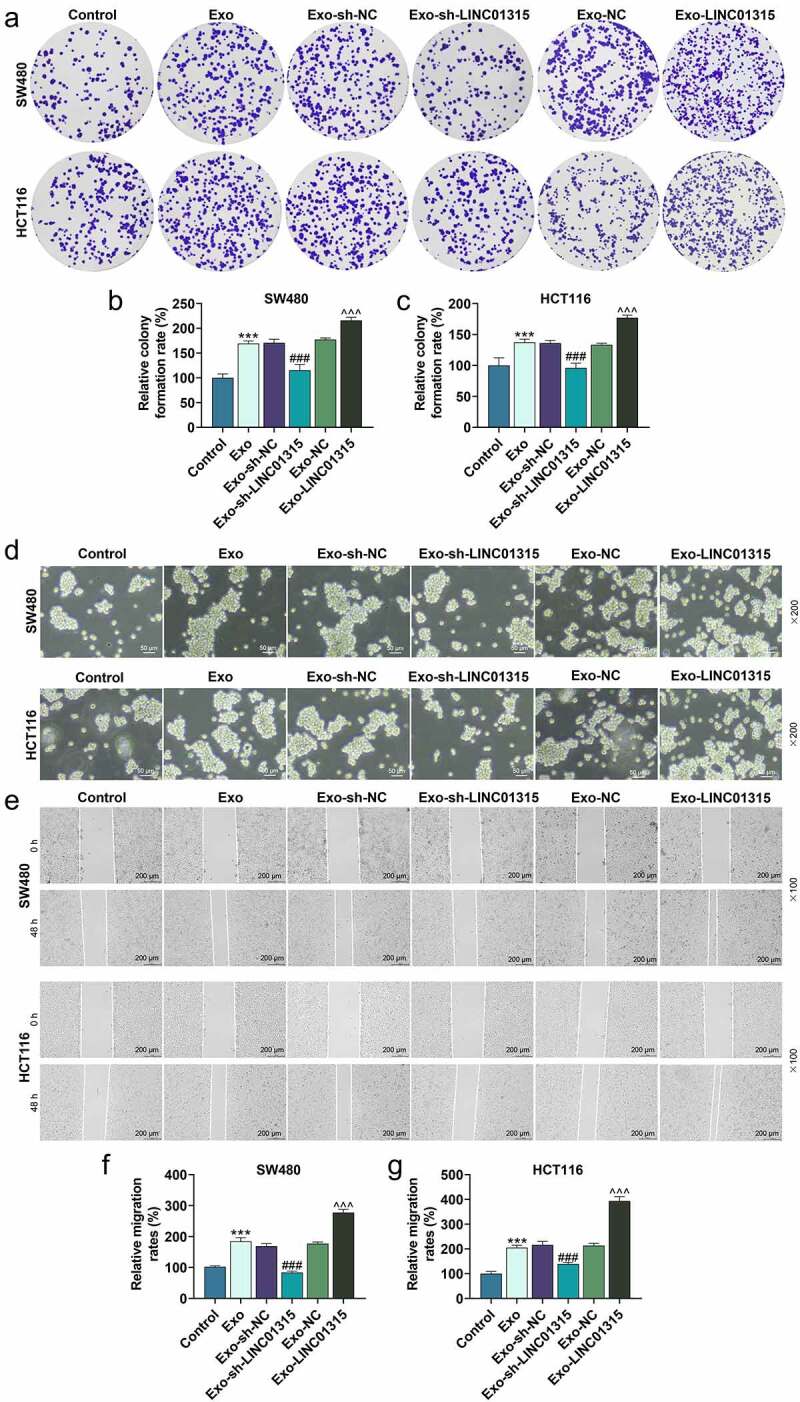
Exosomes derived from CD133^+^/CD44^+^ colorectal cancer cells regulated the stemness of CD133^−^/CD44^−^ colorectal cancer cells.

**Figure 6.**
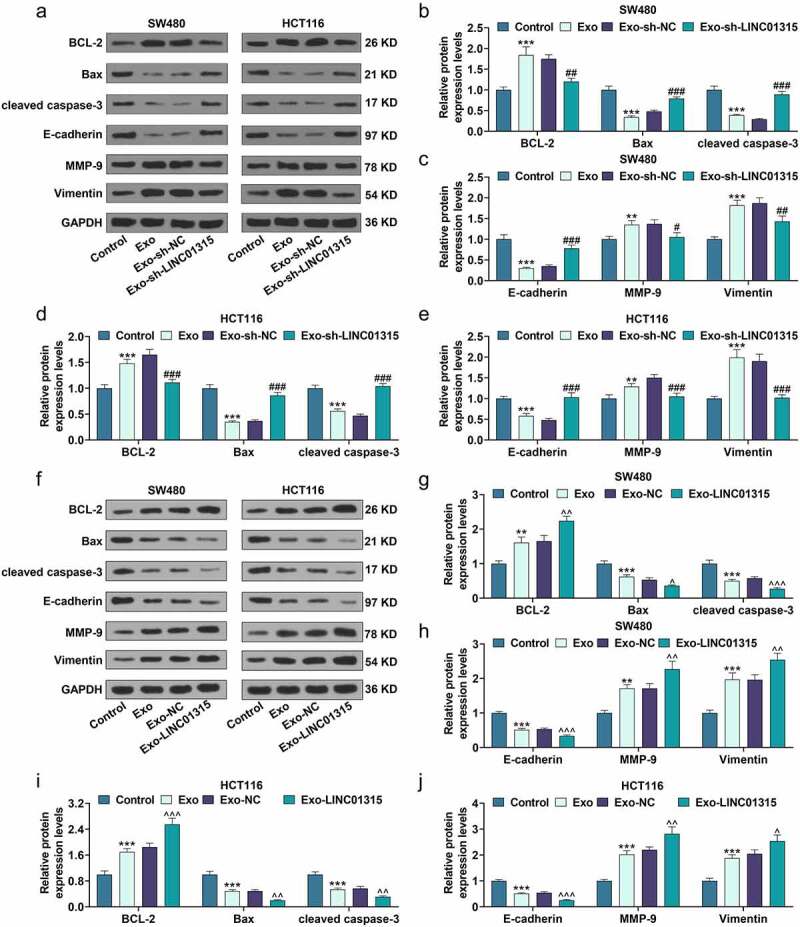
Exosomes derived from CD133^+^/CD44^+^ colorectal cancer cells regulated the expressions of BCL-2, Bax, cleaved caspase-3, E-cadherin, MMP-9, and Vimentin of colorectal cancer cells.

Subsequently, the viability, proliferative, sphere formation, and migratory ability of the SW480 and HCT116 cells after being cultured with exosomes were then evaluated. The results exhibited that exosomes derived from CD133^+^/CD44^+^ colorectal cancer cells elevated the viability (*P* < 0.001, Figure 4(d,e)), proliferation rate (*P* < 0.001, Figure 5(a–c)), sphere formation ability (Figure 5(d)) and migration rate (*P* < 0.001, Figure 5(e–g)). However, compared with the Exo-shNC group, the viability (*P* < 0.05, Figure 4(d,e)), proliferation rate (*P* < 0.001, Figure 5(a–c)), sphere formation ability (Figure 5(d)), and migration rate (*P* < 0.001, Figure 5(e–g)) of SW480 and HCT116 cells were decreased in the Exo-shLINC01315 group. Moreover, compared with the Exo-NC group, the viability (*P* < 0.05, Figure 4(d,e)), proliferation rate (*P* < 0.001, Figure 5(a–c)), sphere formation ability (Figure 5(d)), and migration rate (*P* < 0.001, Figure 5(e–g)) of SW480 and HCT116 cells were increased in the Exo-LINC01315 group. To further verify the results above at the molecular level, the protein expressions of relative factors were then detected through western blot. As illustrated in Figure 6(a–e), exosomes derived from CD133^+^/CD44^+^ colorectal cancer cells up-regulated BCL-2, MMP-9 and Vimentin, while down-regulating Bax, cleaved caspase-3 and E-cadherin in colorectal cancer cells, as compared with those in the control group (*P* < 0.01). Additionally, exosomes derived from CD133^+^/CD44^+^ colorectal cancer cells after being transfected with sh-LINC01315 down-regulated BCL-2, MMP-9, and Vimentin, whereas up-regulated Bax, cleaved caspase-3, and E-cadherin in colorectal cancer cells (*P* < 0.05). As shown in Figure 6(f–j), exosomes derived from CD133^+^/CD44^+^ colorectal cancer cells after being transfected with LINC01315 up-regulated BCL-2, MMP-9, and Vimentin, whereas down-regulated Bax, cleaved caspase-3, and E-cadherin in colorectal cancer cells (*P* < 0.05). All these discoveries demonstrated that CD133^+^/CD44^+^ colorectal cancer cell-derived exosomes containing a high level of LINC01315 the stemness of colorectal cancer cells.

(a-c) The proliferative abilities of colorectal cancer cells after the co-culture with exosomes derived from corresponding CD133^+^/CD44^+^ colorectal cancer cells were evaluated by colony formation assay. (D) The sphere formation abilities of colorectal cancer cells after co-cultivation with exosomes derived from corresponding CD133^+^/CD44^+^ colorectal cancer cells were analyzed by spheroid assay. (e-g) The migratory abilities of colorectal cancer cells after co-culture with exosomes derived from corresponding CD133^+^/CD44^+^ colorectal cancer cells were assessed by wound healing assay. Control group: SW480 and HCT116 cells were normally cultured for 24 h. Exo group: SW480 and HCT116 cells were cultivated with exosomes derived from corresponding CD133^+^/CD44^+^ SW480 and CD133^+^/CD44^+^ HCT116 cells for 24 h, respectively. Exo-sh-NC group: SW480 and HCT116 cells were incubated with exosomes derived from corresponding CD133^+^/CD44^+^ SW480 and CD133^+^/CD44^+^ HCT116 cells which were transfected with sh-NC for 24 h, respectively. Exo-sh-LINC01315 group: SW480 and HCT116 cells were cultured with exosomes derived from corresponding CD133^+^/CD44^+^ SW480 and CD133^+^/CD44^+^ HCT116 cells which were transfected with sh-LINC01315 for 24 h, respectively. Exo-NC group: SW480 and HCT116 cells were incubated with exosomes derived from corresponding CD133^+^/CD44^+^ SW480 and CD133^+^/CD44^+^ HCT116 cells which were transfected with NC for 24 h, respectively. Exo-LINC01315 group: SW480 and HCT116 cells were cultured with exosomes derived from corresponding CD133^+^/CD44^+^ SW480 and CD133^+^/CD44^+^ HCT116 cells which were transfected with LINC01315 for 24 h, respectively. ***P < 0.001, vs. Control; ^###^*P* < 0.001, vs. Exo-sh-NC;^^^^*P* < 0.01, ^^^^^*P* < 0.001, vs. Exo-NC (shNC: negative control for short-hairpin RNA, Exo: exosomes).

(a-j) The expressions of BCL-2, Bax, cleaved caspase-3, E-cadherin, MMP-9, and Vimentin in colorectal cancer cells after the co-culture with exosomes derived from corresponding CD133^+^/CD44^+^ colorectal cancer cells were evaluated by western blot. Control group: SW480 and HCT116 cells were normally cultured for 24 h. Exo group: SW480 and HCT116 cells were incubated with exosomes derived from corresponding CD133^+^/CD44^+^ SW480 and CD133^+^/CD44^+^ HCT116 cells for 24 h, respectively. Exo-sh-NC group: SW480 and HCT116 cells were cultivated with exosomes derived from corresponding CD133^+^/CD44^+^ SW480 and CD133^+^/CD44^+^ HCT116 cells which were transfected with sh-NC for 24 h, respectively. Exo-sh-LINC01315 group: SW480 and HCT116 cells were cultured with exosomes derived from corresponding CD133^+^/CD44^+^ SW480 and CD133^+^/CD44^+^ HCT116 cells which were transfected with sh-LINC01315 for 24 h, respectively. Exo-NC group: SW480 and HCT116 cells were incubated with exosomes derived from corresponding CD133^+^/CD44^+^ SW480 and CD133^+^/CD44^+^ HCT116 cells which were transfected with NC for 24 h, respectively. Exo-LINC01315 group: SW480 and HCT116 cells were cultured with exosomes derived from corresponding CD133^+^/CD44^+^ SW480 and CD133^+^/CD44^+^ HCT116 cells which were transfected with LINC01315 for 24 h, respectively. **P < 0.01, ***P < 0.001, vs. Control; ^##^*P* < 0.05, ^##^*P* < 0.01, ^###^*P* < 0.001, vs. Exo-sh-NC; ^^^*P*< 0.05, ^^^^*P* < 0.01, ^^^^^*P* < 0.001, vs. Exo-NC (shNC: negative control for short-hairpin RNA, Exo: exosomes).

## LINC01315 was predicted to be positively correlated with DPEP1, KRT23, ASCL2 and AXIN2, but negatively correlated with DUSP4 in colorectal cancer cells

**Figure 7.**
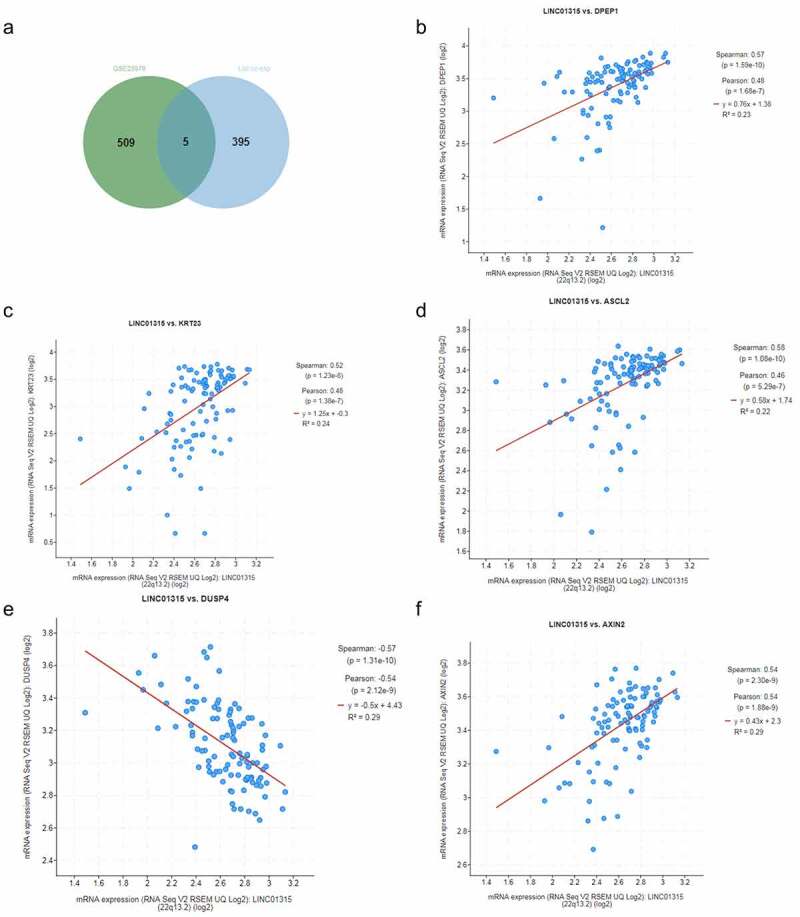
LINC01315 was predicted to be positively correlated with DPEP1, KRT23, ASCL2 and AXIN2, but negatively correlated with DUSP4 in colorectal cancer.

To further find the genes that could interact with LINC01315 in colorectal cancer cells, ciBioportal website was used to predict the co-expression mRNA with LINC01315 in colorectal cancer cells. Meanwhile, GEO analysis (GSE23878 data set) was performed to analyze differentially expressed genes in colorectal cancer cells. After the intersection of these two datasets, we finally found five common genes, namely the Dipeptidase 1 (DPEP1), Keratin 23 (KRT23), Achaete scute-like 2 (ASCL2), AXIN2, and dual-specificity phosphatase 4 (DUSP4) (Figure 7(a)). Besides, in colorectal cancer, positive correlation was found between the expression of LINC01315 and those of DPEP1 (Figure 7(b)), KRT23 (Figure 7(c)), ASCL2 (Figure 7(d)) and AXIN2 (Figure 7(f)), while negative correlation was discovered between the expressions of LINC01315 and DUSP4 (Figure 7(e)), indicating the effects of LINC01315 in colorectal cancer might be associated with the above-mentioned five mRNAs.

(a) The ciBioportal website was used to predict the co-expression mRNA with LINC01315 in colorectal cancer cells. Meanwhile, GEO analysis (GSE23878 data set) was used to analyze the differentially expressed genes in colorectal cancer. From the two data above, we finally found five common genes, which were DPEP1, KRT23, ASCL2, AXIN2, and DUSP4. (b-f) Through ciBioportal website prediction, positive correlations have been discovered between LINC01315 and DPEP1 expressions (b), KRT23 (c), ASCL2 (d), and AXIN2 (f) in colorectal cancer, while a negative correlation was found between LINC01315 and DUSP4 expressions (e). (DPEP1: Dipeptidase 1, KRT23: Keratin 23, ASCL2: Achaete scute-like 2, DUSP4: dual specificity phosphatase 4).

## Discussion

CD133, also known as prominin-1, is a five-transmembrane glycoprotein principally expressed on the cell surface [22]. CD44 is also a member of cell surface glycoprotein family, which is closely related to the proliferation, adhesion, and metastasis of cancer cells [23]. Both CD133 and CD44 proteins are the key markers for a subset of human colorectal cancer stem cells [24]. Therefore, in this research, CD133^+^/CD44^+^ colorectal cancer stem cells were sorted and collected. Cancer stem cells show unlimited self-renewal capacity, thereby possessing the potential to induce tumorigenesis and resistance to chemotherapy drugs [12,13]. In the present study, we discovered that CD133^+^/CD44^+^ colorectal cancer cells had higher viability and stronger abilities of proliferation, sphere formation, and migration than CD133^−^/CD44^−^ and colorectal cancer cells, which was consistent with the discovery of Kim et al. found in Caco-2 cells [14].

LINC01315 is a newly recognized lncRNA, which is abnormally expressed in thyroid cancer, oral squamous cell carcinoma and breast cancer, and can promote the development of these cancers [8–10]. Besides, Liang et al. have also found that LINC01315 is highly expressed in colorectal cancer and LINC01315 silencing can suppress the biological activities of colorectal cancer cells [11]. This research discovered the up-regulation of LINC01315 in CD133^+^/CD44^+^ colorectal cancer stem cells for the first time, indicating that LINC01315 might be associated with the stemness of colorectal cancer cells. In recent years, research about the modulation of lncRNA on stemness of cancer cells is not rare. For instance, lncRNA MACC1-AS1 is demonstrated to promote stemness and chemoresistance in gastric cancer [25]. LncRNA KB-1980E6.3 maintains the stemness of breast cancer stem cells by interacting with IGFBP2 [26]. Additionally, the stemness of cancer cells in colorectal cancer can be regulated by lncRNA FARAS1, LINC00657, HOTAIR, and BCAR4 [27–30]. In our research, LINC01315 silencing was proved to inhibit the viability as well as the abilities of proliferation, sphere formation, and migration of CD133^+^/CD44^+^ colorectal cancer stem cells, demonstrating that LINC01315 played an important role in the stemness of colorectal cancer cells. Nevertheless, how LINC01315-mediated stemness of colorectal cancer cells regulated the development of colorectal cancer still needs further exploration.

RNA molecules, including mRNA, miRNA, circRNA, as well as lncRNA, are highly enriched in exosomes, which are the 30–150 nm vesicles secreted by most cell types [17]. LncRNA MALAT1 shuttled by BMSC-derived exosomes inhibits osteoporosis [31] and lncRNA AFAP1-AS1 contained in exosome enhances resistance to trastuzumab [32]. Given the findings that LINC01315 was enriched in the exosomes isolated from CD133^+^/CD44^+^ colorectal cancer stem cells in this study, we suspected that exosomes containing LINC01315 secreted by CD133^+^/CD44^+^ colorectal cancer stem cells promoted the malignant behaviors of colorectal cancer cells to enhance the development of colorectal cancer. Besides, the cancer stem cell-derived exosomes have also been demonstrated to yield regulatory effects on diverse cancers. For example, the exosomes derived from thyroid cancer stem cells promote metastasis of thyroid cancer by shuttling lncRNAs [20], and glioma stem cell-derived exosomes containing LINC01060 can enhance the progression of glioma [33]. Notably, this research also discovered that the abilities of proliferation, sphere formation, and migration of cancer cells were promoted by colorectal cancer stem cell-derived exosomes, but were inhibited by exosomes derived from colorectal cancer stem cells transfected with sh-LINC01315. Epithelial–mesenchymal transition (EMT) process is one of the molecular mechanisms involved in metastasis of cancer cells [34,35]. The characteristics of EMT are the loss of epithelial surface markers, most notably E-cadherin, and the acquisition of mesenchymal markers including N-cadherin and Vimentin. E-cadherin and Vimentin are frequently dysregulated in multiple human cancers [35,36]. MMP-9 is a key protease to remodel and degrade extracellular matrix, and it has been reported that MMP-9 plays a vital role in tumor development and progression and participates in EMT process [37,38]. Defects in the physiological mechanisms of apoptosis may contribute to different human diseases like cancer [39], pro-apoptotic (Bax and cleaved caspase-3) and anti-apoptotic proteins (BCL-2) that play important roles in the regulation of cell death [40]. In this study, colorectal cancer stem cell-derived exosomes promoted BCL-2, MMP-9 and Vimentin expressions, but inhibited those of E-cadherin, Bax and cleaved caspase-3. Moreover, silenced LINC01315 reversed these effects of exosomes on the above proteins. Our assumption that exosomes containing LINC01315 secreted by CD133^+^/CD44^+^ colorectal cancer stem cells could promote the malignant behaviors of colorectal cancer cells to enhance the development of colorectal cancer was confirmed.

Considering that lncRNA can play its role in various diseases by interacting with mRNA [26], bioinformatics analysis was applied in this research to find the mRNA that could interact with LINC01315 in colorectal cancer. The results demonstrate that down-regulation of DUSP4 is related to the liver and lung metastasis of colorectal cancer [41]. DPEP1 is a marker for the transition from low-grade to high-grade intraepithelial neoplasia and is proved to be an adverse prognostic factor in colorectal cancer [42]. KRT22, a kind of acidic type I keratins, is highly expressed in colorectal cancer, and promotes the proliferation of colorectal cancer cells and the telomerase reverse transcriptase expression in colorectal cancer [43,44]. As a basic helix-loop-helix transcription factor, ASCL2 promotes the self-renewal ability of colorectal cancer progenitor cells [45]. AXIN2 is a negative regulator that suppresses GSK3β-mediated degradation of Snail1 and is proved to exert a promotive effect on colorectal cancer [46]. From bioinformatics analysis, positive correlations were discovered between the expressions of LINC01315 with those of DPEP1, KRT22, ASCL2, and AXIN2, while a negative correlation was found between the expressions of LINC01315 and DUSP4, indicating the effects of LINC01315 in colorectal cancer might be realized by interacting with these genes. However, the experiments designed for verifying the interaction between LINC01315 and these genes were not included in the present study, this is the limitation of this study. Hence, related studies should be established in the future.

## Conclusion

Collectively, the present study discovers that CD133^+^/CD44^+^ colorectal cancer stem cell-derived exosomal LINC01315 promotes the proliferation, migration, and stemness of colorectal cancer cells, providing novel biomarkers and targets for the research and treatment of colorectal cancer to some extent.

## Supplementary Material

Supplemental Material

## Data Availability

The analyzed data sets generated during the study are available from the corresponding author on reasonable request.
