# Supplementary figures and images for: Colorectal cancer stem cell-derived exosomal long intergenic noncoding RNA 01315 (LINC01315) promotes proliferation, migration, and stemness of colorectal cancer cells
Source: Bioengineered. 2022 Apr 26;13(4):10827–42. doi: 10.1080/21655979.2022.2065800 (PMC9161962; doi:10.1080/21655979.2022.2065800)

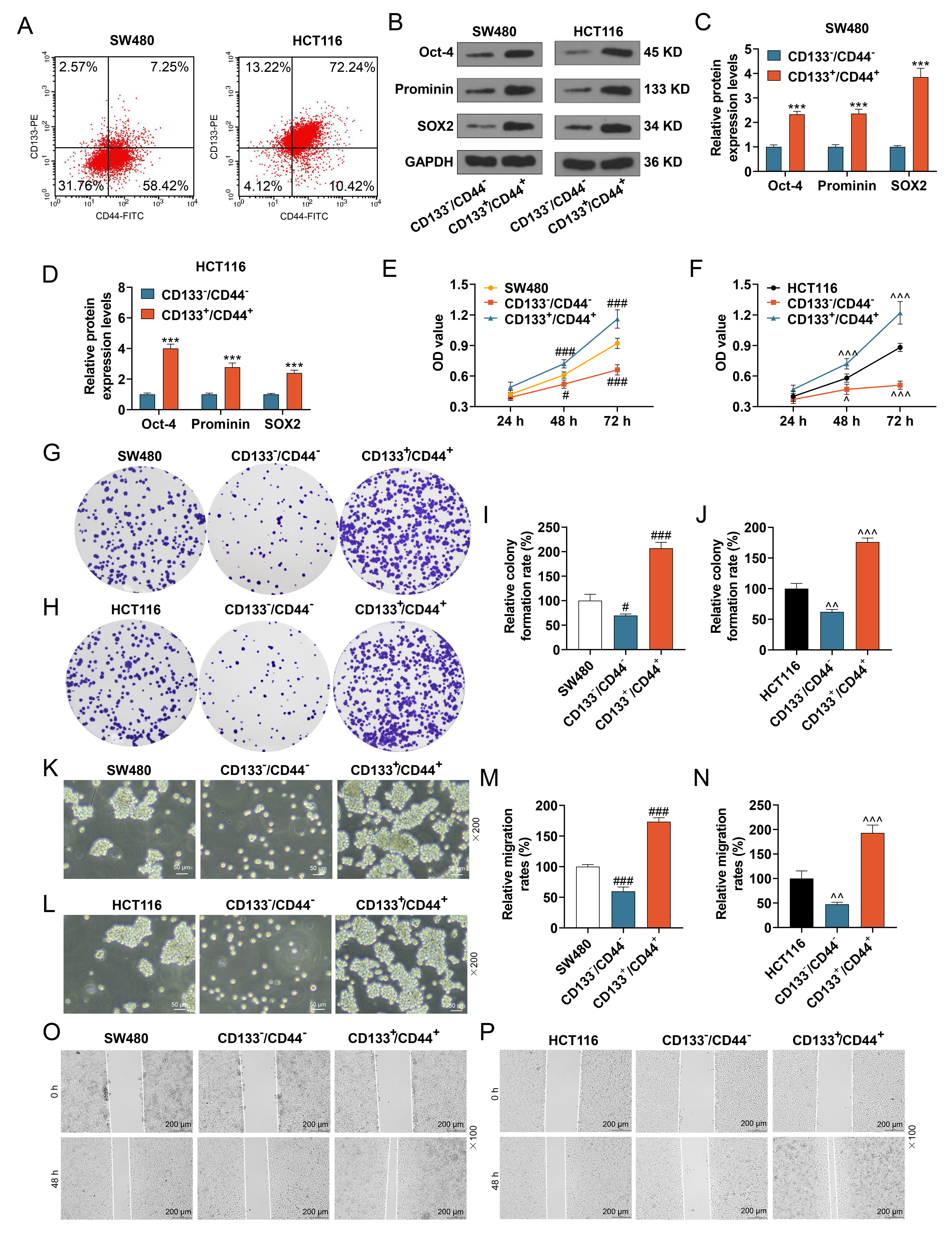

Supplement: Supplemental Material [file KBIE_A_2065800_SM2123.tif]
